# Development of a Novel Dual-Layer Janus Membrane via NIPS Process for Sweep Gas Membrane Distillation (SGMD) and Its Orientation-Dependent Response

**DOI:** 10.3390/membranes16060204

**Published:** 2026-06-10

**Authors:** Ali Sallakh Niknejad, Ananda Pokhrel, Somenath Mitra

**Affiliations:** Department of Chemistry and Environmental Science, New Jersey Institute of Technology, Newark, NJ 07102, USA; as4576@njit.edu (A.S.N.); ap2864@njit.edu (A.P.)

**Keywords:** dual-layer, orientation-dependent, SGMD, superhydrophobicity, desalination

## Abstract

Dual-layer membranes can offer significant advantages in desalination via membrane distillation (MD) compared to conventional single-layer designs. In this study, we report the development of a novel dual-layer nylon/polyvinylidene fluoride (PVDF) membrane with a Janus architecture, specifically engineered for application in sweeping gas membrane distillation (SGMD). The non-solvent induced phase separation (NIPS) method was used to cast PVDF solution on the top of a commercial nylon membrane. Water contact angle (WCA) measurements showed asymmetrical wettability. Scanning electron microscopy (SEM) confirmed that the PVDF layer was firmly anchored to the nylon support without signs of delamination. Desalination experiments were conducted using SGMD, where a significant flux enhancement as high as 81.2% was observed when the feed solution contacted the hydrophilic nylon surface while the hydrophobic PVDF surface faced the permeate side with gas flow. This enhancement was attributed to the high partitioning coefficient of the liquid–vapor mixture on the hydrophilic feed surface and the rapid vapor release across the hydrophobic permeate surface. Overall, these results demonstrate that hydrophilic membranes with small pore sizes (i.e., 0.22 µm) can serve effectively as supports when fabricated using the NIPS process, enabling new configurations for high-performance SGMD.

## 1. Introduction

Membrane distillation (MD) is increasingly recognized as a promising desalination technology due to nearly complete salt rejection [1]. MD offers lower fouling propensity compared to conventional reverse osmosis (RO) and is capable of treating hypersaline waters [2,3]. As a membrane contactor, MD offers several advantages, including the potential to integrate waste heat for energy savings and the ability to operate under mild temperature and pressure conditions [4,5,6]. The process is driven by a vapor pressure gradient across a hydrophobic membrane, which selectively allows water vapor to permeate while retaining dissolved non-volatile solutes [7,8]. Depending on the configuration, the vapor can be collected in different ways: in direct contact MD (DCMD), it condenses into a cold stream [9]; in vacuum MD (VMD), a vacuum enhances vapor extraction [10]; in air gap MD (AGMD), an air layer separates the membrane from the condensation surface [11]; and in sweep gas MD (SGMD), an inert gas sweeps vapor to an external condenser [12].

A critical factor for MD membranes is surface hydrophobicity, typically measured by water contact angle (WCA). Various fabrication techniques, such as electrospinning, non-solvent induced phase separation (NIPS), templating, etc., have been used to engineer hydrophobic and superhydrophobic surfaces [13,14,15]. Considerable effort has focused on combining nanoscale roughness with nanoparticle coatings, yielding superhydrophobic membranes with WCAs exceeding 150° [16,17,18,19,20,21].

Recent research has introduced dual-layer membranes, combining a hydrophobic and a hydrophilic layer, into DCMD and AGMD systems. These structures can reduce mass transfer resistance, increase flux, improve thermal efficiency, lower temperature polarization, and enhance both mechanical strength and wetting resistance in treating challenging waters. While promising, reports remain limited, and fabrication has largely relied on electrospinning and co-extrusion. For example, Tijing et al. [22] developed dual-layer PVDF-HFP/PAN nanofibrous membranes via electrospinning, while Bonyadi and Chung [4] and Wang et al. [23] prepared hydrophobic/hydrophilic hollow fibers by co-extrusion, finding that thinner hydrophobic layers improved permeability. Hou et al. [24] deposited an electrospun layer onto commercial PTFE membranes for anti-fouling in saline oily waters, and later [25] fabricated PTFE-supported composites. Despite these advances, both methods have limitations in scalability for reasons such as weak interlayer adhesion due to use of two polymers with different chemistries, potential delamination, and complex fabrication methods using multi-step fabrication processes, leaving opportunities for simpler approaches such as NIPS to produce novel dual-layer membranes with desirable properties.

SGMD is unique among MD configurations because the permeate side consists of a flowing sweep gas rather than a liquid or vacuum environment. This minimizes heat loss, reduces temperature polarization, and can lower mass transfer resistance compared to DCMD, VMD, or AGMD [26,27,28,29]. Since the sweep gas carries vapor to an external condenser, SGMD is also particularly attractive for solvent recovery applications [30]. The presence of two flowing streams liquid feed and sweep gas introduces distinct mass transfer resistances governed by the kinetics of each stream, which creates opportunities to optimize dual-layer membrane orientation for higher flux and efficiency. Unlike DCMD and VMD, SGMD performance is strongly influenced by sweep gas flow rate, which can be coupled with feed temperature and flow rate to further enhance permeability while maintaining high salt rejection.

The objective of this study was to develop a dual-layer Janus membrane for SGMD and to systematically evaluate how membrane orientation affects its performance. In addition, we aimed to establish a simple, scalable fabrication method by casting a wet film of a binary PVDF/NMP solution onto a commercial nylon substrate to form the Janus structure. This approach leverages the hydrophilicity, diverse available pore sizes and mechanical robustness of the nylon support, which, to the best of our knowledge, has not previously been utilized in the casting of MD membranes.

## 2. Materials and Methods

### 2.1. Materials and Chemicals

PVDF powder (Freflon^®^FR205; white powder; standard relative density, 1.76~1.79 g/cm^3^; melt flow rate, 4–8 g/10 min; rotational viscosity, ≥60 Pa.s; intrinsic viscosity, 10^2^ mL/g; melting point, 168–175 °C; thermal decomposition, 370 °C) was bought from Jiangsu FreChem Co., Ltd., Jiangning District, Nanjing, China. According to the provided technical data sheet, this grade is suitable to make water treatment membranes. A supported commercial hydrophilic nylon (0.22 µm pore size) membrane was bought from Tisch Scientific, Cleves, OH, USA. N-methyl-2-pyrolidone (NMP) solvent was bought from J.T. Baker (Phillipsburg, NJ, USA). Anhydrous isopropyl alcohol (IPA) was bought from Florida Laboratories, Inc., Fort Lauderdale, FL, USA. Table salt was bought from a local supplier to make feed solutions. Deionized (DI) water was produced using a Barnstead 5023 water purification system (Thermo Fisher Scientific, Dubuque, IA, USA).

### 2.2. Dual-Layer Membrane Fabrication

First, 2.75 g of PVDF powder with 7.25 g of NMP solvent was stirred for 20 h at 80 °C. The cast solution was placed in an oven for another 5 h at 80 °C to remove the air bubbles as thoroughly as possible. Air bubbles in the casting solution can create defects like small holes on the membrane surface. A PVDF wet film was cast on nylon membrane using a casting blade with a gap thickness of 300 µm and was then immersed in IPA for at least 30 min. Then it was transferred into another container containing fresh IPA: DI (1:1) solution to remove the residual NMP solvent and avoid shrinkage. The dual-layer membranes were dried in air before use. The membranes were labeled as hydrophobic (HFB)/hydrophilic (HPL) and HPL/HFB according to the feed flow circulation. For example, when feed meets the PVDF layer, it is called HFB/HPL format, and vice versa, it is referred to as HPL/HFB.

### 2.3. Characterization

A scanning electron microscope (SM-7900F, JEOL USA Inc., Peabody, MA, USA) was used to take images of the membranes. Membrane samples were fractured using liquid nitrogen to provide cross-sectional SEM images. Membranes were platinum coated using a sputter coating device (Quorum Q150V Plus Automatic Coater, Quorum Technologies Ltd., Lewes, UK) before taking any images. Water contact angle (WCA) of the membranes was measured using a WCA testing device (Ramehart Model 250, ramé-hart Instrument Co., Succasunna, NJ, US). A 5 µL DI water droplet was gently dropped on the membrane surface using a micropipette (Acura 825 Single-Channel Pipette, Socorex Isba SA, Ecublens, Switzerland). Mean values of at least three measurements were reported. Thickness of the membranes was measured and reported using cross-sectional SEM images. Liquid entry pressure of water (LEP) was measured using a homemade set-up. A plastic membrane module (Hangzhou Cobetter Filtration Equipment Co., Ltd., Hangzhou, China) was used to hold the membranes. A dispensing pressure vessel (1 gal, Sigma Aldrich, St. Louis, MO, USA) was used to pressurize DI water (1 L) using a nitrogen gas cylinder. The pressure where the first water droplet was detected was regarded as the LEP value (see Figure S1). The overall porosity of the membranes was measured using a gravimetric method. Round membrane samples were cut using a leather circle cutter (2.5 cm in diameter). The samples were then kept in vacuum oven at 50 °C for 24 h to remove the moisture, and were subsequently immersed in IPA for another 24 h. Overall porosity was measured using the equation below [31]:

ε = (m_wet_ − m_dry_)/*ρAδ* × 100 (1)

where m_wet_ and m_dry_ are the wet and dry membrane weight (g); ρ, A and δ are the density of IPA (0.786 g/cm^3^), membrane area (cm^2^) and thickness (cm), respectively.

### 2.4. SGMD

**Figure 1:**
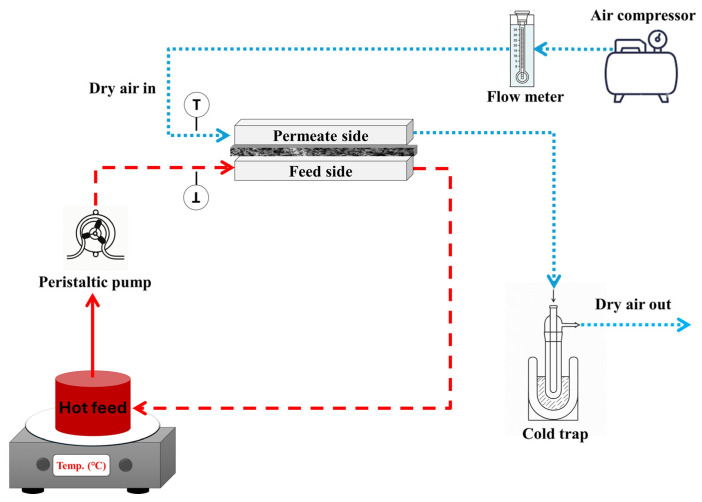
Schematic diagram of the SGMD system.

A lab-made device was used to run SGMD tests (see Figure 1). Feed water was heated using a magnetic stirrer hot plate connected to a temperature sensor (FOUR E’S SCIENTIFIC, Guangzhou, China). Feed temperature was kept at 70 °C by the sensor. Inlet and outlet feed and permeate temperatures were recorded using a set of thermometers (K-type, Cole Parmer Instrument Company, Vernon Hills, IL, USA) and mean value reported. Mean value for 70, 60 and 50 °C of heater was recorded as 62 ± 0.1, 53 ± 1 and 42 ± 0.1 °C, respectively. Air flow was kept at 3 L/min throughout the tests, except for the investigation of air flow rate effects on SGMD performance. The effective area of the module was 0.00145 m^2^ (with an effective diameter of 4.3 cm). The feed flow rate was fixed using a peristaltic pump (Cole Parmer, Instrument Company, Vernon Hills, IL, USA). A counter-current flow regime was used. The mean values for the inlet and outlet sweep gas temperatures were recorded at 27 ± 1 for 70 °C, 26.5 ± 1 for 60 °C and 25.5 ± 1 for 50 °C when the air flow rate and feed flow rate were set at 3 L/min and 65 g/min. Then, a 10 g/L sodium chloride (NaCl, table salt) solution was used to conduct desalination tests. Conductivity of the feed and collected water in the permeate side was measured using a portable pH/electrical conductivity (EC) meter (PC 400, Apera Instruments, Columbus, OH, USA). A conductivity–concentration (mg/L) graph was plotted to calculate the salt rejection factor (see Figure S2). The permeate side was prewashed with DI water before conducting SGMD. The EC of the permeate was measured after the collected permeate had been circulated though the permeate side using DI water-washed tubes to ensure that no salt leakage was present on the bottom side of the membrane. As there is no water circulation on the permeate side, salt deposition on the bottom side of the membrane was possible, which could potentially cause inaccuracies in determining wetting. The permeate flux was calculated using the weight of the water loss in the feed side divided by the time interval (h) and membrane effective area.

The evaporation efficiency (EF) was calculated using the formula below:

EF (%) = (J A ∆H_v_/Q_m_) × 100

where J, A, ∆H_v_ and Q_m_ represent the flux, membrane effective area, heat of vaporization and membrane heat transfer, respectively. The mass transfer coefficient (MTC) was calculated by determining the flux and vapor pressure for the feed (P_f_) and permeate (P_p_) as follows:

MTC (kg/m^2^.Pa.s) = J/(P_f_ − P_p_)

## 3. Results

### 3.1. Membrane Characterization

**Figure 2:**
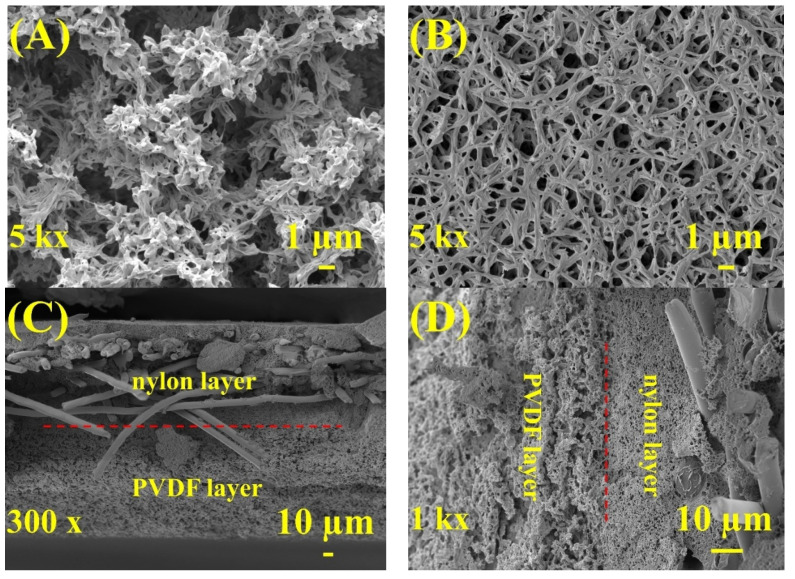
SEM images of the dual-layer membrane. Red dotted line shows the interface between PVDF and nylon layers: (**A**) PVDF layer, (**B**) nylon layer and (**C**,**D**) cross-sectional images.

The dual-layer membranes were successfully fabricated using the method described above. Their morphology, including the hydrophobic PVDF (HFB) surface, the hydrophilic nylon (HPL) surface, and the cross-sectional structure, is shown in Figure 2. The HFB layer exhibits a porous, fibrous-like structure with a somewhat entangled appearance due to the use of isopropanol (IPA) as the non-solvent. In contrast, water as a strong non-solvent does not allow for gradual phase separation, leading to a more uniform surface morphology. When a milder non-solvent is employed, crystallization-controlled phase separation produces a bi-continuous structure [32,33]. This structure resembles lichens, with a highly branched, sponge-like texture that helps trap more air beneath the membrane. For comparison, SEM images of the commercial nylon membrane (HPL) also show its porous and fibrous structure.

Cross-sectional SEM images of the dual-layer membrane reveal a distinct interfacial layer, marked by dotted lines. The thick fibers originate from the nylon support, as confirmed by the manufacturer’s report. Layer thicknesses measured from SEM images (Table 1) show that the HFB layer is 113 ± 5 µm and the HPL layer is 103 ± 4 µm, giving the total dual-layer thickness of 221 ± 5 µm. The relative contributions of the layers were calculated as 51.13% for HFB and 46.61% for HPL layers.

The overall porosity of the dual-layer membrane was measured as 62.1% ± 2 (Table 1), which falls within the recommended literature range of 30–85% [14,34]. High porosity enhances permeability and reduces conductive heat loss. The slightly lower porosity of the dual-layer membrane is likely due to the nylon support, where thick fibers reduce the porosity.

WCA measurements revealed that the contact angle for the HFB layer was as high as 154.3° ± 1.7. Membrane durability in the desalination process can be improved using a superhydrophobic membrane by limiting the salt intrusion into the pores. As seen, the water droplet on the surface of the PVDF top layer beaded up and formed a WCA higher than 150°. In contrast, the WCA on the HPL part was measured as 36.7° ± 2.57, showing that the nylon side is hydrophilic. To show how hydrophobic the PVDF part is, a video (see Video S1) shows the behavior of DI water at the time of contact with the membrane surface. Also, another defining feature of a superhydrophobic surface—the ability of water droplets to rebound or bounce—is shown in Video S2.

The LEP value of the dual-layer membrane was measured as 124 ± 6 kPa (see Table 1), which is comparable to commercial polytetrafluoroethylene (PTFE) and PVDF membranes. Deka et al. [35] reported LEP of 130 kPa for a Millipore brand PVDF membrane with a 0.45 µm pore size. In another review paper, the LEP values for a commercial PTFE membrane with 0.45 and 0.5 µm pore sizes were reported as 138 and 124 kPa, respectively. Also, a LEP of 103 kPa was reported for the Millipore PVDF with a pore size of 0.45 µm [36].

### 3.2. Orientation-Dependent SGMD

**Figure 3:**
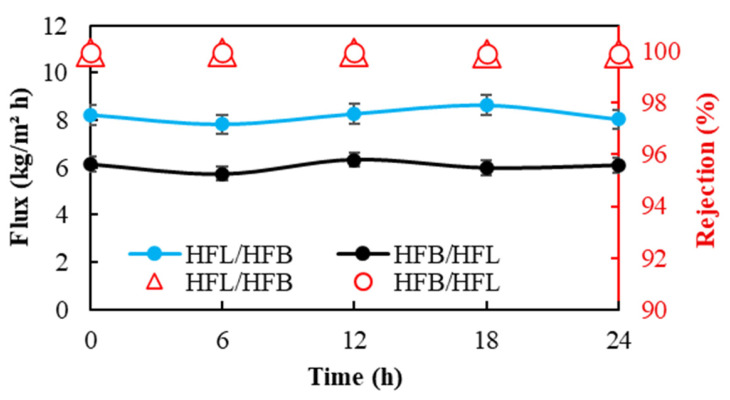
SGMD performance of the dual-layer when feed meets the hydrophilic and hydrophobic layers of the membrane. Feed temperature, 62 ± 0.1 °C; feed flow rate, 65 g/min; SGMD duration, 24 h.

Desalination performance in the SGMD process was evaluated by directing the feed through either the HFB or HPL side of the dual-layer membrane. Interestingly, higher flux was obtained when the feed contacted the hydrophilic side (or HPL/HFB mode) compared to the hydrophobic side (or HFB/HPL mode). In the HFB/HPL configuration, the flux was 6.06 ± 0.7 kg/m^2^.h, whereas in the HPL/HFB configuration, it increased to 8.20 ± 1.1 kg/m^2^.h, corresponding to a flux enhancement of 35.3% (Figure 3).

**Figure 4:**
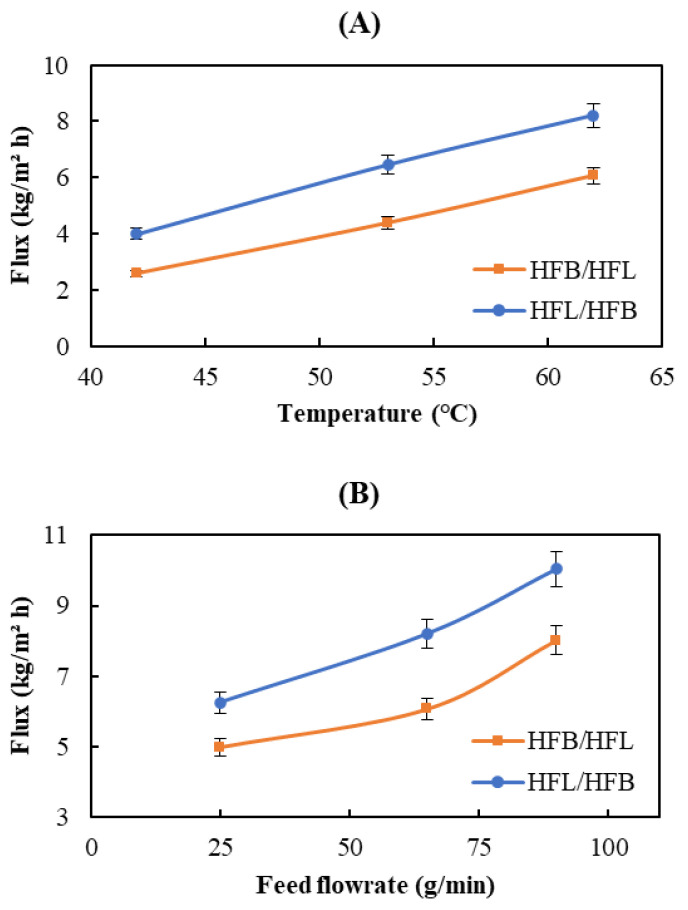
(**A**) Feed temperature and (**B**) feed flow rate effect on membrane performance. Feed temperature, 62 ± 0.1 °C, except for temperature effect investigation on SGMD performance; feed flow rate, 65 g/min, except investigation of feed flow rate on SGMD performance; SGMD duration, 6 h; air flow rate, 3 L/min.

**Figure 5:**
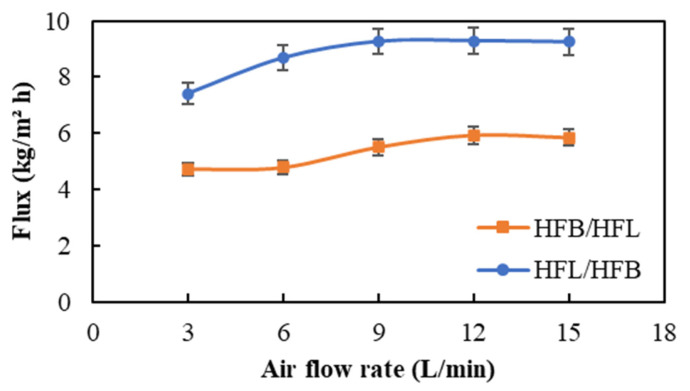
Flux as a function of air flow rate in the HFB/HPL and HPL/HFB modes at a feed temperature of 53 ± 0.1 °C and feed flow rate of 65 g/min.

Salt rejection remained above 99% in both orientations, confirming stable separation performance regardless of feed direction. The membranes also demonstrated long-term operational stability. Each membrane was tested for at least 72 h in total, including 24 h desalination runs for each orientation and short-term experiments to assess performance under different operational conditions. This is shown in Figure 3, Figure 4 and Figure 5. All SGMD trials were conducted in concentration mode, without replenishing the feed tank with make-up water.

The effects of feed temperature and flow rate on permeate flux are shown in Figure 4. As expected, higher feed temperature increased vapor pressure across the membrane, thereby enhancing the driving force for permeation. For example, flux in the HFB/HPL or PVDF/nylon configuration increased from 2.57 to 6.06 kg/m^2^.h as the feed temperature rose from 42 ± 0.1 °C to 62 ± 0.1 °C, while in the HPL/HFB or nylon/PVDF configuration it increased from 3.99 kg/m^2^.h to 8.20 kg/m^2^.h in the same temperature range. Similarly, increasing the feed flow rate further enhanced flux due to the reduction in polarization effects. At 62 ± 0.1 °C, SGMD tests conducted for 6 h at flow rates of 25, 65, and 90 g/min consistently showed this improvement.

The effect of air flow rate on desalination performance via SGMD was examined for both HPL-HFB and HFB-HPL orientations over a range of 3–15 L/min. As anticipated, increasing air flow enhanced permeate flux by reducing the boundary layer, thereby increasing the driving force. At higher flow rates, the concentration at the membrane surface approached bulk concentration, indicating reduced concentration polarization because of improved mass transfer [12,37]. The flux variation with air flow rate is shown in Figure 5. In the HPL/HFB (nylon/PVDF) configuration, flux was increased as high as 81.24%. Notably, between 2 and 15 L/min, the overall percentage flux enhancement was nearly identical for both orientations, measured as 25.29% for PVDF/nylon and 25.27% for nylon/PVDF. Beyond 12 L/min, both modes reached a plateau.

MTCs are summarized in Table 2. The MTC increased with air flow rate in both configurations and reached a maximum value of 2.28 kg/m^2^.Pa.s in the HPL/HFB mode. In addition, average evaporation efficiency was higher in the HPL/HFB configuration, showing an enhancement of about 20% rising from an average of 60.80% to 80.49% compared to that of the PVDF/nylon system. This suggests that both air flow and membrane orientation contribute synergistically to improved permeability.

### 3.3. Mechanism of Water Permeation in the HFB/HPL and HPL/HFB Modes

**Figure 6:**
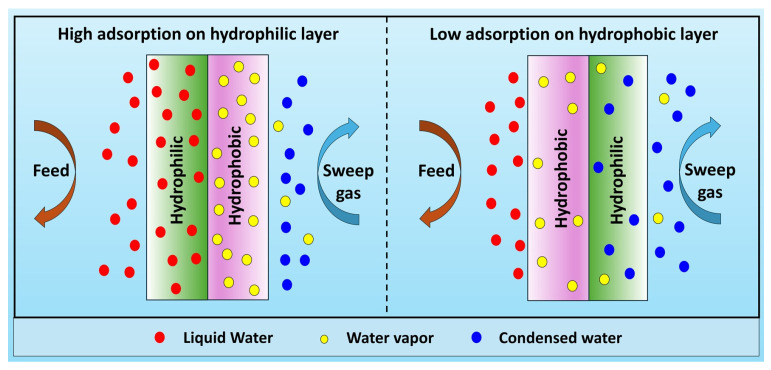
Mechanism of water purification through the HFL/HFB membrane.

The mechanism of water vapor permeation in SGMD involves adsorption of the liquid–vapor mixture on the feed side, transport through the membrane via the sorption–diffusion model, and subsequent evaporation on the permeate side. Figure 6 illustrates the potential role of nylon/PVDF layers in enhancing permeate flux. When the hydrophilic layer is placed on the feed side, more water–vapor mixtures adsorb onto the membrane surface, thereby increasing both the effective partition coefficient and the available surface area. In contrast, if the hydrophobic layer faces the feed side, water is repelled, reducing partitioning of water molecules onto the membrane surface. On the permeate side, if the hydrophilic layer is present, permeated water vapor adsorbs strongly, making evaporation more difficult and leading to higher mass transfer resistance. Conversely, with the hydrophobic layer on the permeate side, the permeated vapor is readily repelled, lowering mass transfer resistance. The results are similar to what has been presented by Cong and Guo [38], who fabricated PAN nanofiber dual-layer membranes via electrospinning, maintaining a constant thickness of 60 µm, to evaluate desalination performance using the AGMD process. They reported that the hydrophilic layer, once wetted by condensed vapor, can induce temperature polarization and secondary vaporization, further increasing mass transfer resistance.

Overall, flux improvement in dual-layer membranes particularly in SGMD, arises from the combined effects of enhanced partitioning on the feed side and reduced mass transfer resistance on the permeate side.

## 4. Conclusions

A PVDF cast solution was formed on the commercially available nylon membrane to fabricate dual-layer with asymmetric wettability using the NIPS process. A milder non-solvent, i.e., IPA, was used to make a sponge-like and rough PVDF layer to represent superhydrophobicity. Water droplets did not show any tendency to attach to the surface of the PVDF layer in the dual-layer membrane and bounced several times following impact, highlighting a superhydrophobic surface characteristic. Using a saline solution, permeate flux and salt rejection of the fabricated membranes were evaluated from both sides. Salt rejection was always above 99%. Permeate flux was measured to be 35.3% higher when feed met the nylon layer. With a higher air flow rate, the maximum enhancement reached 81.24% when feed faced the nylon layer. The mass transfer coefficient and thermal efficiency also showed the same trend.

This study is particularly noteworthy, as there have been no prior reports on the use of dual-layer Janus membranes in the SGMD process. The present findings clearly demonstrate that membrane orientation plays a critical role in SGMD performance. Overall, dual-layer membrane architecture offers several advantages in SGMD and holds strong potential for expanding its applicability to broader areas such as solvent recovery, where SGMD provides distinct operational benefits. Future efforts could focus on enhancing wetting resistance through the incorporation of advanced materials into multi-layer configurations. For example, introducing nanomaterials as intermediate layers to create a tailored sandwich structure may simultaneously improve liquid entry pressure (LEP) and overall SGMD performance. Such strategies could enable the design of more robust, high-functionality membranes for next-generation SGMD applications.

## Figures and Tables

**Table 1:** Physical characteristics of the dual-layer membrane.

Layers	Thickness (µm)	Overall Porosity (%)	WCA (°)	LEP (kPa)
**PVDF layer**	113 ± 4	62.1 ± 2	154.3 ± 1.7	124 ± 6
**Nylon layer**	103 ± 4		36.7 ± 2.57	

**Table 2:** Mass transfer coefficient at different air flow rates at 53 ± 0.1 °C; feed flow rate, 65 g/min.

Membrane	MTC (kg/m^2^ Pa s)				
	Air Flow Rate (L/min)				
	3	6	9	12	15
**PVDF/Nylon**	1.13	1.15	1.32	1.4	1.39
**Nylon/PVDF**	1.85	2.17	2.27	2.33	2.28
**Nylon/PVDF**	**Enhancement (%)**				
	63.72	88.70	71.97	66.43	64.03

## Data Availability

Data will be available upon request.

## Supplementary Materials

The following supporting information can be downloaded at https://www.mdpi.com/article/10.3390/membranes16060204/s1, Figure S1: Schematic diagram showing the component of home-made LEP device; Figure S2: EC-concentration graph to calculate salt rejection; Video S1: Water droplet behavior in contact with the PVDF side of the dual-layer membrane; Video S2: Water droplet bouncing on the top PVDF layer.
