# Supplementary material for: Development of a Novel Dual-Layer Janus Membrane via NIPS Process for Sweep Gas Membrane Distillation (SGMD) and Its Orientation-Dependent Response
Source: Membranes (Basel). 2026 Jun 10;16(6):204. doi: 10.3390/membranes16060204 (PMC13304111; doi:10.3390/membranes16060204)
Supplement: Supplementary file 1 [file membranes-16-00204-s001.zip › membranes-4170544-supplementary.pdf]

Development of a Novel Dual-Layer Membrane via NIPS Process for Sweep Gas Membrane Distillation (SGMD) and Its Orientation Dependent Response

Ali Sallakh Niknejad, Ananda Pokhrel, Somenath Mitra *

Department of Chemistry and Environmental Science, New Jersey Institute of Technology, Newark, New Jersey 07102, United States

Corresponding author: Somenath Mitra (Email: somenath.mitra@njit.edu)

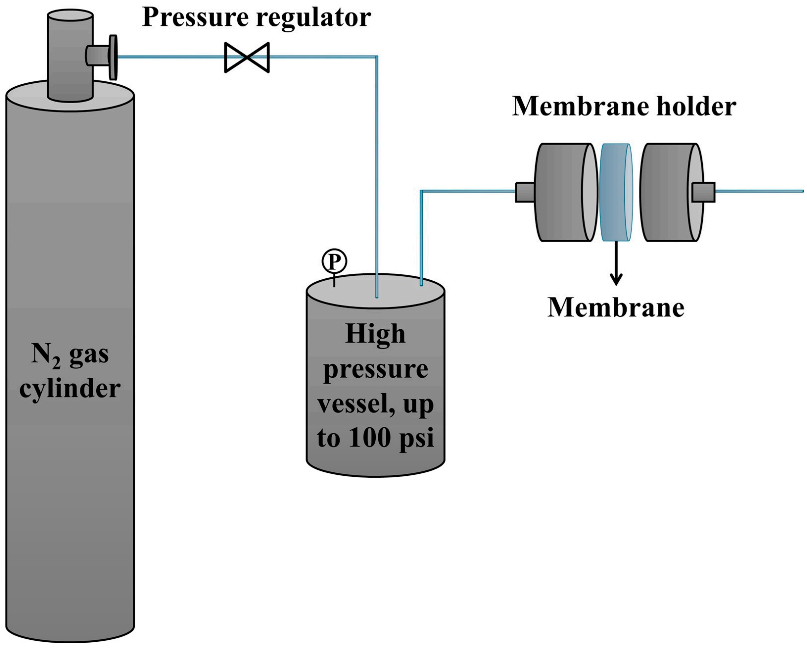

Figure S1. Schematic diagram showing the component of home-made LEP device.

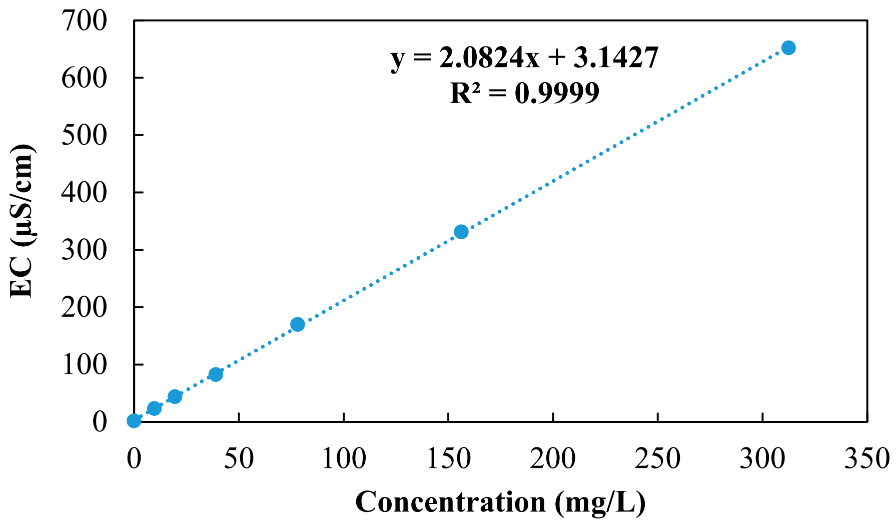

Figure S2. EC-concentration graph to calculate salt rejection.

Untitled video -
Made with Clipchamp

Video S1. Water droplet behavior in contact with the PVDF side of the dual-layer membrane.

Bouncing video.mp4

Video S2. Water droplet bouncing on the top PVDF layer.
